# New Appliances and Things Medical

**Published:** 1906-06-30

**Authors:** 


### NEW APPLIANCES AND THINGS MEDICAL.

**[We shall be glad to receive, at our Office, 28 & 29 Southampton Street, Strand, London, W.C., from the manufacturers, specimens of all new preparations and appliances which may be brought out from time to time.]**

### FUSSELL AND CO.'S PREPARATIONS.

(London: 4 Monument Street, E.C.; and Norway. Prepared in England.)

The condensed milks of this firm are well known, and we have now received from them three preparations consisting of condensed full cream milk with pure coffee, pure cocoa, and pure chocolate. These preparations are termed Black, Violet, and Brown Butterfly brand respectively. We have tried these three products, and find them palatable and convenient. It must be noted that they are fairly rich in sugar, and hence are unsuitable for diabetic or glycosuric patients. With this single reservation, however, we find them excellent. They keep for a considerable time after being opened, and have practically a uniform strength. One dessert-spoonful being required to make a breakfast cupful of the finished beverage, the diluent being water.

